# Overrepresentation of *APOE* ε4 carriers in genome‐wide association studies of memory function and memory decline

**DOI:** 10.1002/alz.13824

**Published:** 2024-04-22

**Authors:** Md Shafiqur Rahman

**Affiliations:** ^1^ MRC Biostatistics Unit University of Cambridge Cambridge Biomedical Campus Cambridge UK; ^2^ Department of Clinical Neurosciences University of Cambridge Cambridge Biomedical Campus Cambridge UK

Archer et al. reported a cross‐ancestry genome‐wide association study (GWAS) on memory performance (*n* = 27,633) and cognitive decline (*n* = 22,365), using data from four aging cohorts.^1^ Associations were adjusted for age, sex, and the first five principal components. The study of this nature is rare and important for understanding cognitive performance and its relation to neurodegenerative diseases. However, by design, Archer et al.’s^1^ study had some limitations that are important to address during the analysis phase. Alzheimer's disease (AD) has a protracted pre‐clinical period,^2^ which makes studying memory decline challenging, especially when the study sample is predominantly composed of individuals at older ages (considering age‐related cognitive decline), and those at risk of AD are overrepresented in the study sample compared to the general population prevalence. In the study, the percentage of apolipoprotein E (*APOE*) ε4 carriers in the four participating cohorts ranged from 26.16% to 46.41%, with participants having a baseline mean age > 72. Study samples also included individuals with mild cognitive impairment and AD.^1^ Consequently, the emergence of the *APOE* locus as a significant determinant for baseline memory performance and memory decline was not surprising and might be predominantly driven by the inclusion of a higher proportion of *APOE* ε4 carriers who are at high risk of dementing illness.^3^ This might also explain why the authors observed a stronger genetic correlation with AD than with cognitive performance and educational attainment, suggesting that the phenotypes primarily capture aspects of AD rather than memory performance or dementia‐related memory decline that precedes the diagnosis of AD. Additionally, sensitivity analysis was not conducted after adjusting for *APOE* allele carriers or removing ε4 carriers. Considering these limitations and absence of proper replication, caution should be exercised when interpreting the purported roles of prioritized genes (e.g., *SLC25A44*, *BSX*, and *DPP8*) in age‐ and dementia‐related memory decline.

## CONFLICT OF INTEREST STATEMENT

The author reports no conflicts of interest. Author disclosures are available in the supporting information.

## FUNDING INFORMATION

Alzheimer's Society, Grant/Award Number: AS‐PG‐18b‐022; National Institute for Health and Care Research (NIHR) BioResource.

## CONSENT STATEMENT

Not required.

## Supporting information

Supporting Information
